# Perspective of older people on which medicines they need to report to healthcare professionals as part of a medicines history: a qualitative descriptive study

**DOI:** 10.1007/s11096-025-01890-7

**Published:** 2025-03-20

**Authors:** Emily Griffin, Kenneth Lee, Christopher Etherton-Beer, Joon Soo Park, Amy Page

**Affiliations:** 1 Discipline of Pharmacy, School of Allied Health, The University of Western Australia, Perth, Australia; 2 Monash Rural Health, Monash University, Bendigo, Australia; 3 Medical School, The University of Western Australia, Perth, Australia

**Keywords:** Aged, Attitude, Medication therapy management, Medication reconciliation, Polypharmacy

## Abstract

**Background:**

The medicines that older people consider relevant as part of their medicine regime may be reflected in their reporting of medicines to healthcare professionals during a medicine history.

**Aim:**

This study aimed to understand the perspectives of people, aged 65 or older, on which medicine(s) they reported as part of their medicine regimen.

**Method:**

A qualitative descriptive study was conducted in Australia using semi-structured interviews to investigate the perspectives of which medicines were reported in a medicines history by people aged 65 years or older, taking at least one medicine. Participants were recruited until data saturation was reached. The interviews were audio recorded, transcribed, and thematically analysed using the Framework Method.

**Results:**

Six main themes emerged from sixteen participants: reporting medicines that solved a medical condition, medicines recommended by healthcare professionals, regular medicines, route of administration, combination products, and multiple tablets, doses, or part doses. Participants’ beliefs and experiences impacted whether they included a medicine in their regimen. Participants inconsistently reported infrequent medicines, varying formulations, and multiple doses. Non-oral and over-the-counter medicines were commonly included if a healthcare professional recommended them. In contrast, supplements were seldom included.

**Conclusion:**

This study highlights the variation between participants’ perspectives in reporting medicines, suggesting that older people’s self-reporting of medicines is generally inconsistent. Our findings encourage clinicians to specifically enquire about medicines for acute health conditions, medicines commonly not prescribed by healthcare professionals, irregular medicines, and non-oral medicines to improve reporting by older people, to obtain the Best Possible Medication History.

**Supplementary Information:**

The online version contains supplementary material available at 10.1007/s11096-025-01890-7.

## Impact statements

- Older people’s perspectives of medicine-related burden and polypharmacy are currently unclear, and the practise implications of this are potentially important due to the varied methods of counting and reporting medicines. The variation in how older people, healthcare professionals, and researchers report medicines highlights the need to consider this variation when taking medicine histories and considering medicine burden.
- This study found variation in how older people report medicines based on their beliefs and experiences of medicines and healthcare. In particular, there are inconsistencies in how older people report infrequent medicines, varying formulations, and multiple doses of the same medicine within the same day. Inconsistent reporting of medicines by older people could lead to inaccurate reporting of medicines and inaccurate interpretation of medicine-related burden, polypharmacy, and medicine management.
- This study identified participants reported combination products, simultaneous multiple units, and part-units as a single medicine, warranting quantitative investigation to confirm this finding.
- Educating clinicians about the variation in the types of medicines older people report may assist with highlighting the importance of detailed history-taking by clinicians to obtain the Best Possible Medication History.

## Introduction

Medicines included in a Best Possible Medication History (BPMH) should include over-the-counter, prescription, traditional and complementary medicines to obtain the most complete and accurate medicine list, to reduce the risk of medicine-related harm[1]. A BPMH includes dosing frequency, number of administration times, and number of units (e.g. tablets) of medicines. However, there is inconsistency in research and practise when reporting medicines included in a medicine regimen such as interpreting multiple units of the same medicine administered simultaneously, multiple doses of the same medicine throughout the day, irregular medicines (e.g. *pro ne rata* medicines), combination tablets, duration of use, and non-oral medicines[1–5]. It is well-documented that taking multiple medicines (polypharmacy) is associated with an increased risk of medicine-related adverse events including drug-drug interactions, drug-disease interactions, adverse effects, administration errors, reduced quality of life, non-adherence, increased morbidity, mortality, and cost [1, 2, 6–9]. It is unclear which medicines are reliably reported in medicine regimens. Given there is no standardised approach to the medicines included by healthcare professionals (HCPs) or researchers, there is likely variation in older people’s perspectives about which medicines to report to HCPs.

A systematic review found older people’s beliefs around polypharmacy and the influence of these underexplored [10]. Another recent study found patients are not actively involved in polypharmacy management [11]. One study identified medicine regimens become more complex with an increasing number of medicines, and regimen complexity contributes to high levels of medicine-burden, challenges with medicine management, and impacts the daily life of older people and carers. Additionally, the burden of complex regimens is often hidden from practitioners. Participants in this example had polypharmacy (defined as more than five medicines) or “a complex regime” (if taking less than five medicines), but, it was unclear who counted the medicines (participants or researchers), and which medicines were included such as irregular, parenteral, complementary therapies [12]. Another study investigating medicine-related burden found each an increasing number of medicines and dosing frequency increased medicine burden [13]. The number of medicines was self-reported and again it is unclear which medicines were included [13]. A questionnaire about medicine burden (Living with Medicines Questionnaire) describes the burdensome domains by “users of long-term medicines” [14]. The number of medicines were reported by participants which included *pro ne rata*, other infrequent medicines, and “other formulations”, however, it is unclear how medicines taken more than once per day were counted, or if non-prescription medicines were included [14]. The focus of such studies surrounds the burden of medicines, but not the medicines included in this burdensome regime.

Studies have shown a relationship between older people’s beliefs about medicines and medicine burden, medicine adherence, and a possible relationship between their beliefs and polypharmacy, and inappropriate medicine use [10]. So, it is anticipated individual perspectives will weigh into their decision-making to include or exclude a medicine in their count. Currently no studies are available to show this. Considering this, the medicines included in current studies is likely inconsistent and potentially misrepresentative of the true count and burden. Ambiguity in medicine count and reporting in current studies could mean participants were taking more than reported, therefore, interpreting the burden and polypharmacy perspectives by older people, in terms of medicine quantity, becomes unclear.

Guidance on good practice for HCPs around medicine in England is based on four key principles; understanding the older person’s experience, medicine choice is evidence-based, medicine use is as safe as possible, and medicine optimisation is part of routine practice [15]. The medicines standard in Australia, recommends older people and carers are provided with sufficient information about their medicines to make informed decisions and achieve adherence [8]. Guiding older people about medicine-related questions to ask their HCPs, and allowing opportunity to ask for information and advice can improve older people’s experience, their health outcomes, and possibly avert errors [8]. To align with patient-centred care and good practice, identifying which medicines patients include in their regime may allow for a deeper understanding of older people’s perceived medicine burden and which medicines they may reliably report to HCPs. This may inform clinician education about the medicines they may reliably report to ensure clinician medicine history taking is detailed, precise, and reliable.

### Aim

This study aimed to understand the perspectives of older people, aged 65 or older, on which medicines they reported in a medicine history. Identifying which medicines are included may improve clinicians’ understanding of the medicines older people may reliably report in their interactions.

### Ethics approval

Approval for this study was obtained from the University of Western Australia Human Research Ethics Committee (HREC 2022/ET000988).

## Method

### Study design

This qualitative descriptive study implemented semi-structured interviews to investigate the perspectives of which medicines were reported in a medicines history by people older than 65. This study is reported in line with the Consolidated Criteria for Reporting of Qualitative Research (COREQ) checklist.

### Participants

Participants were eligible to participate if they were 65 years or older and taking at least 1 medicine. Participants were excluded if they did not speak English. Potential participants were recruited directly and indirectly via emailed invitations through a professional organisation (Pharmaceutical Society of Australia), placement of advertisement posters in pharmacies, residential aged care facilities, and social media (LinkedIn, Facebook, Instagram).

### Setting

The semi-structured interviews were conducted between August and September 2023, either online via audio or video conference (using Microsoft Teams) or in person at a mutually agreed-upon location.

### Data collection

The interview guide included questions asking participants about reporting and counting prescription, non-oral (e.g. patches, creams, injections), over-the-counter Schedule 2 (‘Pharmacy Medicines’) and Schedule 3 (‘Pharmacist Only Medicines’) and complementary medicines (e.g. vitamins, supplements). These questions were open-ended in design and relevant prompts were formulated to gain insight into each participant’s perspective on which medicines they included as part of their medicine regimen (Appendix 1).

Data were collected using audio recordings on 2 separate devices. The participant demographics were collected. Responses to questions were documented in the interview guide. Each interview lasted approximately 20 minutes.

Audio recordings were named ‘Participant [number]’ to prevent identification. Any identifying details were removed during transcription.

Semi-structured interviews were conducted until data saturation; when no new codes or themes emerged. This was determined by authors after listening to the interviews, cross-checking the code book (Appendix 2) and collectively deciding that no new themes were emerging in at least 2 consecutive interviews. No repeat interviews were conducted. Transcripts were not returned to participants for comment or correction.

### Data analysis and quality assurance

All semi-structured interviews were transcribed verbatim using an online transcription software (Otter.ai, Los Altos, California, USA). All transcripts were manually checked for accuracy by 1 researcher and reviewed by a second researcher if there were uncertainties. Transcripts were imported into NVivo software version 12 (Lumivero, Burlington, Massachusetts, USA) to facilitate data analysis. Data analysis were performed with files named ‘Participant [number]’ to prevent identification.

Data were thematically analysed using the Framework Method [16], and codes were derived inductively. Specifically, 2 researchers developed an initial analytical framework (code book) following independent coding of a single transcript. This framework was applied to subsequent transcripts and updated whenever new codes were identified (final codebook included in Appendix 2). Any adjustments to the code book were noted in the audit trail, detailing changes (Appendix 3). Where different codes were assigned by different coders, these were discussed between coders. Where the different codes were deemed relevant, multiple codes were assigned to the same passage in the transcript. Where agreement on course of action could not be reached between two coders, the senior author was involved to adjudicate. Following completion of the coding process, all codes and associated participant quotes were charted on a matrix to facilitate identifying underlying themes. The codes, themes and subthemes, and their relationships are included in Appendix 4.

To establish trustworthiness, various methods including analyst triangulation, peer debriefing, and negative case analysis were implemented.

## Results

All participants were community-dwelling individuals residing in metropolitan Western Australia. A total of 16 participants were interviewed. Each semi-structured interview lasted approximately 20 min. The demographics of the participants are listed in Table 1. No new codes were generated after the 13th semi-structured interview. Data saturation was confirmed during the 16th interview.

**Table 1:** Participants' demographic information

Total participants, n	16
Age in years (median ± IQR)	77 ± 7.5
*Gender, n*	
Male	8
Female	8
*Cultural Backround, n*	
Australian	7
Indigenous	1
European	3
Malaysian	3
Chinese	2
*Management of medicines, n*	
Manage own medicines, no pill box	10
Manage own medicines, packs own pill box	5
Someone else manages medicines	1

Six themes emerged:

- Reporting medicines because they solve a medical condition or problem;
- Reporting medicines recommended by healthcare professionals;
- Reporting regular medicines;
- Reporting medicines in relation to their route of administration,
- Counting combination products as single medicines;
- Counting multiple tablets or doses or part doses.

### Reporting medicines because they solve a medical condition or problem

Participants commonly included medicines if they believed the medicines managed or solved a health problem. When participants were asked about their regime, some participants included non-oral medicines, for example topical products, or non-prescription medicines because they believed these solved a problem:

*“An itch is a problem. Use medicines to get rid of problems or to alleviate problems. Wouldn't want them for any other reason.”*

[Participant 10]

*“If you have problem with a sore or anything or if you have pain... you apply this (regarding the use of topical medicines)... I think it should be part of the medicine to heal, to get better.”*

[Participant 8]

### Reporting medicines recommended by healthcare professionals

Participants commonly included medicines recommended or prescribed by their HCP; including recommended non-oral and over-the-counter medicines. Supplements were seldom included in the participants’ count.

*“I hadn't considered them as medical because they weren't prescribed. The ones I consider a medicine are the ones that are prescribed by the doctor. The other ones are supplements.”*

[Participant 11]

*“I think for me and my mindset if I'm buying something just over-the-counter myself, I don't really count that as a medication. But if it's something that's been offered to me or prescribed to me by someone with some medical knowledge, like, you know, a pharmacist or a doctor then yes, I probably count that as medication.”*

[Participant 12]

### Reporting regular medicines

Participants commonly included medicines taken regularly; some extended this belief as rationale to include non-prescription medicines.

*“It's okay to say it is included in the* (medicine) *list because I take it regularly every day*.”

[Participant 4]

*“Well, if you have to take it on a regular basis, that means that it's a part of the medicine”*

[Participant 7]

Medicines taken less frequently (less than daily) were often not included in the participants’ medicine regimen, for example when participants were asked about vaccines:

*“Well, suppose of medicine* [sic]* is an ongoing, sort of day-to-day thing, a vaccination is a one-off bit like the COVID vaccination, or the flu injection is a one-off from last 12 months. And it's generally not something you do yourself anyways… medicine is stuff that you administer to yourself on a daily basis.”*

[Participant 15]

### Reporting medicines in relation to their route of administration

Medicines administered orally were consistently included by participants. Participants’ personal beliefs and experiences with non-oral medicines lead to variation in including such medicines:

*“No* (regarding counting injections). *Well, my guess is because they're not a tablet*.... *No* (regarding counting patches) ...*I think I'm gonna* [sic] *think of medicine as being something you take orally. Swallow.”*

[Participant 16]

Formulations administered parenterally or topically, such as injections, infusions, or creams were occasionally included if the participant perceived it offered clear health benefits or if it was administered by an HCP:

*“I think I should be counting the injection as part of the medicine... Infusion, yes.* (regarding counting infusions) *...Because infusion given to you is part of the medicine that you do, to get you better* [sic]. *Yes* (regarding counting creams) ... *Because if you have problem with a sore or anything or if you have pain... you apply this... I think it should be part of the medicine to get better.”*

[Participant 8]

*“Yes* (regarding counting vaccinations)* ...I guess we could call it medication... because I tend to go to the doctor's surgery and the nurse would give me the injection there and I guess it formalises it for me if it's done within the doctor's surgery.”*

[Participant 12]

### Counting combination products as a single medicine

Participants commonly counted combination products as 1 medicine, providing rationale of “1 tablet” or because the combination treats 1 health condition:

*“This is the tablet that I'm prescribed. It has 2 ingredients, but they've got to be taken together in this particular form, combination. So, it's just as far as I'm concerned, 1 medicine.”*

[Participant 10]

*“If it's a combined tablet, but it's treating the same thing, then it's 1 medication because it's doing 1 job.... If you're saying that they're adding 2 things and I'm still taking it as a headache tablet or a pain relief tablet, then I guess it is still to me 1 medication.”*

[Participant 12].

### Counting multiple tablets or doses or part doses

Multiple units (e.g. tablets) of the same medicine taken simultaneously were commonly counted as 1 medicine by participants:

*“Like my prednisolone…I take 3 in the morning these 3 tiny little things, but I consider that as 1 while it has 3 tablets… I suppose I look upon it as a unit… (I was) on four of those (Ursodeoxycholic acid tablets) a day, now I'm on 2 but I just think of it as a unit. I'm on Ursodeoxycholic (acid), that's 1, prednisolone, that's another 1.*

[Participant 9]

Multiple doses of the same medicine throughout the day were identified as 1 medicine by some participants:

*“I would have said 1* (medicine)*. If I can take it 3 times a day, or once a day I'm still on that medicine. So yeah, it depends on how you look at it. If you want to know how many times I take medicines per day, I’ll say 3. But if it was just how many medicines do you take in a day? I would say just 1.”*

[Participant 14]

Some participants counted multiple doses separately; for example, 1 participant counted 3 doses of paracetamol as 3 medicines:

*“Three medicines because it's 3 times a day, I would count that as 3 medicines, 3 doses of medicine.”*

[Participant 14].

Overall, part doses, e.g. half a tablet, were commonly considered 1 medicine by participants.

*“You’re only taking a portion ...Micardis comes in 20 milligrams, 40 milligrams and 80 milligrams, right. Now, sometimes the pharmacy they don't have the 40 milligrams, they only have 80 milligrams. Alright, so, the pharmacist tells you, sir, I don't have 40 milligrams, I'm giving 80, but you can cut it in 2 and so it's 40-40. It's still 1, it's still 1 medication, but you're splitting it. So, as far as you're concerned, it’s still 1 medication. You're cutting the tablets in the 2. That's it.”*

[Participant 6]

## Discussion

### Statement of key findings

Our study aimed to understand the perspectives of people, aged 65 or older, about which medicine(s) should be counted as part of their medicine regimen. Individual beliefs and experiences around medicine intended to solve a health problem, who prescribed or recommended the medicine, frequency, route of administration, formulation, multiple units or doses of the same medicine, and combination products led to diversity in participants’ interpretation of whether a medicine should be included in their medicine count. Combination products, simultaneous multiple units, and part-units were all counted in the same way by participants. In contrast, counting multiple doses, non-oral medicines, and irregular medicines, varied amongst participants. The inconsistency in the way participants reported medicines aligns with available evidence including the inconsistency in reporting medications by HCPs and researchers [1, 5, 17]. There is little information available as to how older people count medicines. One study found older people report a different number of medicines compared with their electronic medical record and the medicines provided by their pharmacy [18]. Our study contributes to research in an underexplored area, providing insight into the beliefs, experiences, perspectives, and the consistencies and inconsistencies when older people count medicines.

### Strengths and weaknesses

Our study’s strengths included utilising various methods to establish trustworthiness. To reduce observer bias, analyst triangulation was utilised; 2 researchers coded each transcript separately. Peer debriefing was also conducted with 2 peers unrelated to the research during analysis to uncover biases or assumptions made by analysts. Additionally, negative case analysis was conducted whenever data contradicted common patterns emerging during analysis. These strategies improved the consistency between analysts and the credibility of the study. Additionally, utilising 1 primary interviewer and an interview guide increased consistency between semi-structured interviews.

The limitations of the study included a predominance of Caucasian and Asian participants. There was no strategy to ensure a balance of cultures and ethnicities thus a variety of methods were used to recruit participants. Due to our study’s limited variation in participant ethnicities and the potential cultural variation in reporting medicines, for example reporting of traditional or complementary medicines, future research could investigate other ethnicities to identify cultural differences in reporting medicines. Additionally, recruitment was based on participants self-reporting using at least 1 medicine, so, participants whom did not consider themselves to be taking medicines by their own definition, were not recruited. Therefore, our study may have excluded those taking few regular or prescribed or irregular medications thus, our findings cannot be transferred to this population.

### Interpretation

Participants tended to report medicines they believed *solved a problem* for them. Indications referenced by participants included acute conditions such as “sore throat” or “cough” which was provided as rationale to include a medicine in their count. Contrastingly, chronic health conditions were not justification to include a medicine, rather, medicines for chronic conditions were reported and counted in relation to the experience of regularity (i.e. daily medicines for chronic conditions) or by the believed necessity of reporting due to prescription/recommendation by an HCP. Regardless of the rationale used, overall, we found participants included medicines for acute *and* chronic health conditions. No evidence is available regarding counting medicines related to acute versus chronic conditions. A person’s perception of illness and perception of illness-burden is related to medicine adherence in hypertension [19]. Thus, the perception of some acute conditions or illness-burden of some acute conditions may contribute to participants’ justification for counting medicines relating to it. Identifying which acute conditions older people believe to be burdensome may assist in determining which medicines they may reliably report to HCPs. Our findings encourage HCPs to ask about medicines in relation to acute conditions during a BPMH.

HCPs recommendations weighed into participants’ contemplations around reporting medicines. HCPs mentioned by participants included doctors, pharmacists, nurses, chiropractors and physiotherapists. Some participants justified including medicines because an HCP recommended them, similarly, others justified omitting a medicine because they were not recommended by an HCP. These beliefs highlight the trust people place in practitioners’ initiation of medicines [12]. Older people place a higher priority on medicines they deem to be of greater importance and have a higher motivation to take medicines as prescribed, which may contribute to their decision-making here [20]. Our findings align with 1 study finding non-oral medicines were counted only if they were prescribed or recommended by a HCP, or, if they provided a health benefit [21]. Thus, people may reliably report medicines recommended by an HCP. We recommend HCPs undertaking a BPMH to ask specifically about medicines not prescribed by an HCP and categories of medications commonly not prescribed by an HCP such as supplements and off-the-shelf medicines.

Participants considered administration frequency when reporting medicines. Some participants believed medicines taken less than daily would not be included in their count. Therefore, infrequent medicines, such as vaccines, even if they could be perceived to solve a health problem, such as creams, were not included by some participants. Studies show poorer compliance to topical products compared to oral agents, particularly over time [22]. Reduced adherence could be an underlying reason for exclusion of topical products by some participants. There is no evidence available regarding counting infrequent medicines in relation to adherence. Other participants believed daily medicines should be included in their count. Counting medicines taken daily highlights the significance of consistent use in older people’s medicines management [12]. One study considered ‘regular medicines’ as those used on a routine basis and *pro re nata* medicines [21]. These results show best-practice may not align with common practice by HCPs and researchers, nor older peoples’ perspectives. Thus, we encourage HCPs obtaining BPMHs to ask specifically about irregular medicines, such as injections, or “as required” medicines.

Medicines administered parenterally (e.g. infusions) and topically (e.g. creams and patches) were reported inconsistently by participants. Generally, parenteral medicines were not included in a participants’ report. Currently, there is little information available around reporting of formulations other than tablets or non-oral administration routes. Similarly, there are gaps in literature surrounding parenteral medicines such as vaccinations, depot injections and implants. Routine is an important component of medicine management for older individuals[12]. Consequently, people may fail to recall irregular medicines, resulting in unreliable reporting to HCPs, therefore, we encourage HCPs to ask specifically about non-orally-administered medicines including infusions, injections, implants, patches, and vaccinations.

Participants usually reported prescribed medicines taken orally. Some participants believed *only* medicines administered orally should be included. Whereas some participants omitted some oral medicines due to their personal exclusion criteria, for example, those not recommended by an HCP. A systematic review highlighted that 88.6% of articles did not specify routes of administration when reporting medicines [5]. A BPMH includes all routes and formulations of medicines, prescribed and not prescribed [1]. Thus, all formulations, regardless of frequency, and source of initiation should be included in a person’s reported or documented medicine regime. Our study highlights varied reporting of all medicines, including oral medicines, highlighting the importance of HCPs asking specific questions during a BPMH.

There was variation in reporting medicines with multiple doses per day. Some participants believed multiple doses should be counted as 1 medicine, whereas others believed multiple doses should be counted separately. A systematic review found the frequency of administration a significant contributing factor to the overall complexity of an individual’s medicine regime. Additionally, older people who do not consider multiple daily dosing as 1 medicine may feel increasingly burdened by the number of units [23]. Identifying how people count their medicine may allow clinicians to better identify people that may experience higher medicine-burden. Interestingly, 1 participant believed their answer to counting multiple doses depended on the question, for example “how many times?” versus “how many medicines?”. This participant counted 3 doses of the same medicine as “1 medicine” in 1 instance and “3 medicines” in another instance. The variation in response reiterates medicine-related communication by HCPs should consider a person’s health-literacy, be easy to understand, tailored to the individual, and collaborative to ensure an accurate message for information gathering and educational purposes [24, 25]. Consequently, we recommend HCPs ask specific, unambiguous, initial questions, and seek follow-up and clarification during a BPMH.

Participants reported combination products, simultaneous multiple units, and part-units as 1 medicine. A systematic review reported 96.1% articles counted combination products as 1 medicine which is consistent with our participants’ perspectives [5]. Research has demonstrated a reduction in pill-burden and improved adherence with combination tablets in older people [26]. Our research supports consideration of combination products for improved medicine management and reduced medicine-burden, since combination products are perceived as 1 medicine. Participants consistently counted simultaneous multiple units, and part-doses, as 1 medicine. No evidence detailing how older people count combination products simultaneous multiple units. Our study provides new insight into how people count these examples and may contribute to future research about older people consistently reporting such examples.

Medicines included in medicine regimes is often incompletely or not described in studies [27]. A study aiming to create a framework for counting medicines identified inconsistency in medicine count by older people when using different methods[27]. Overall, our findings align with the Theory of Planned Behaviour. Our participant’s beliefs and experiences including their attitudes, subjective norm, and perceived behavioural control impacted whether or not they reported a medicine. Our research further solidifies the theory of planned behaviour as a reasonable theory to explain medicine use behaviour, however, these elements and this framework likely only partially explain our participants’ behaviour as shown in other studies [28]. Additionally, self-reporting of medicines is a significant source of information for health services and during transitions of care (TOC) [29]. Researchers and clinicians should be aware of the likely inconsistencies in self-reporting in research and practise, and the potential consequences of such inconsistencies including a misrepresentation of drug burden and polypharmacy, and potential and actual drug interactions and adverse effects. With this awareness, clinicians can ensure detailed history taking to ensure a BPMH aligning with best practice.

### Further research

Further research could be conducted over a broader sample including varied demographics and location. Future research could investigate perspectives around failing to report medicines, particularly those initiated without HCPs’ input, and perspectives around acute versus chronic medicines contributing to medicine count. Additional research could investigate perspectives of medicine reporting at TOC. Our study could be utilised to conduct follow-up studies to investigate the impact of HCP knowledge about variation in reporting medicines on the clinician’s BPMH technique, and to develop a guide for health professionals to better collect self-reported medicines from patients during a medicine history.

## Conclusion

Our study revealed the inconsistencies in how people count regular and irregular medicines, medicines administered via non-oral routes, and formulations other than tablets. Despite people sharing a similar approach to counting combination products, multiple units of the same medicine, and part-doses of medicines, our study highlights the need for clinicians to take detailed medicine histories.

## Supplementary Information

Below is the link to the electronic supplementary material.

Supplementary file1 (DOCX 1695 KB)
