# Supplementary material for: Perspective of older people on which medicines they need to report to healthcare professionals as part of a medicines history: a qualitative descriptive study
Source: Int J Clin Pharm. 2025 Mar 20;47(5):1177–85. doi: 10.1007/s11096-025-01890-7 (PMC12432078; doi:10.1007/s11096-025-01890-7)

Supplementary material

**Appendix** **1** Interview guide


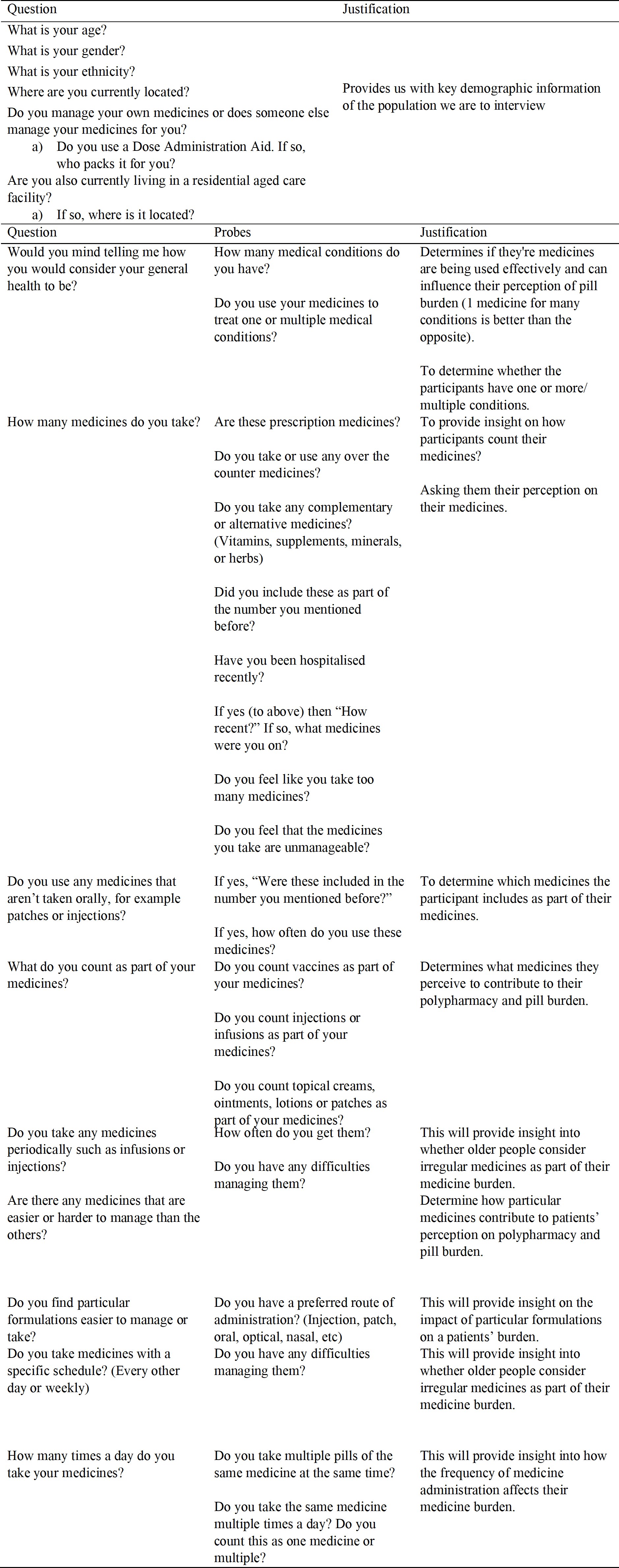


**Appendix 2** Code book


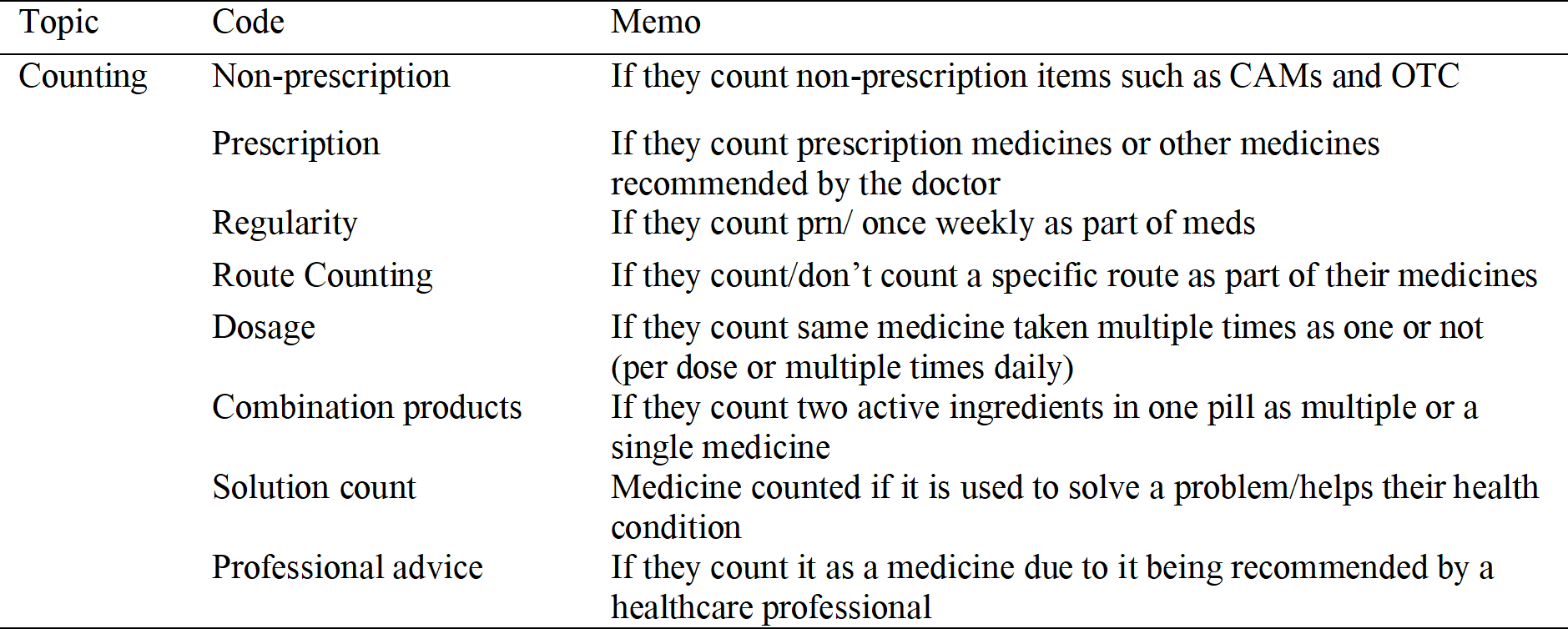


**Appendix 3** Audit trail


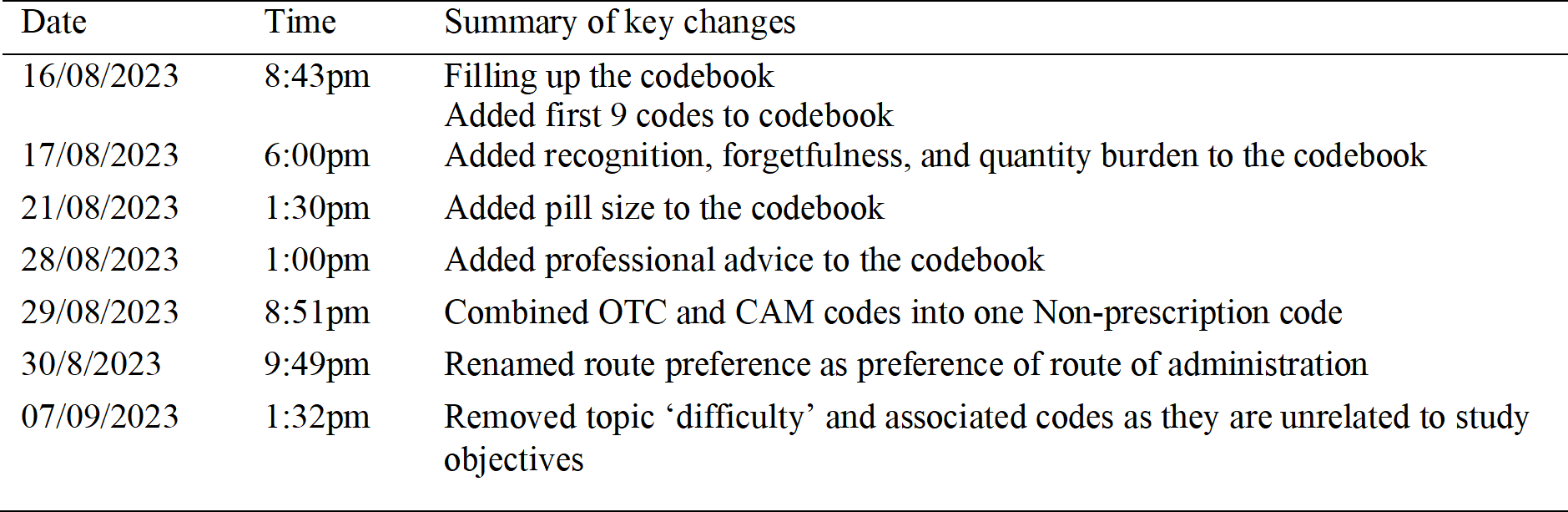


**Appendix 4** Coding tree


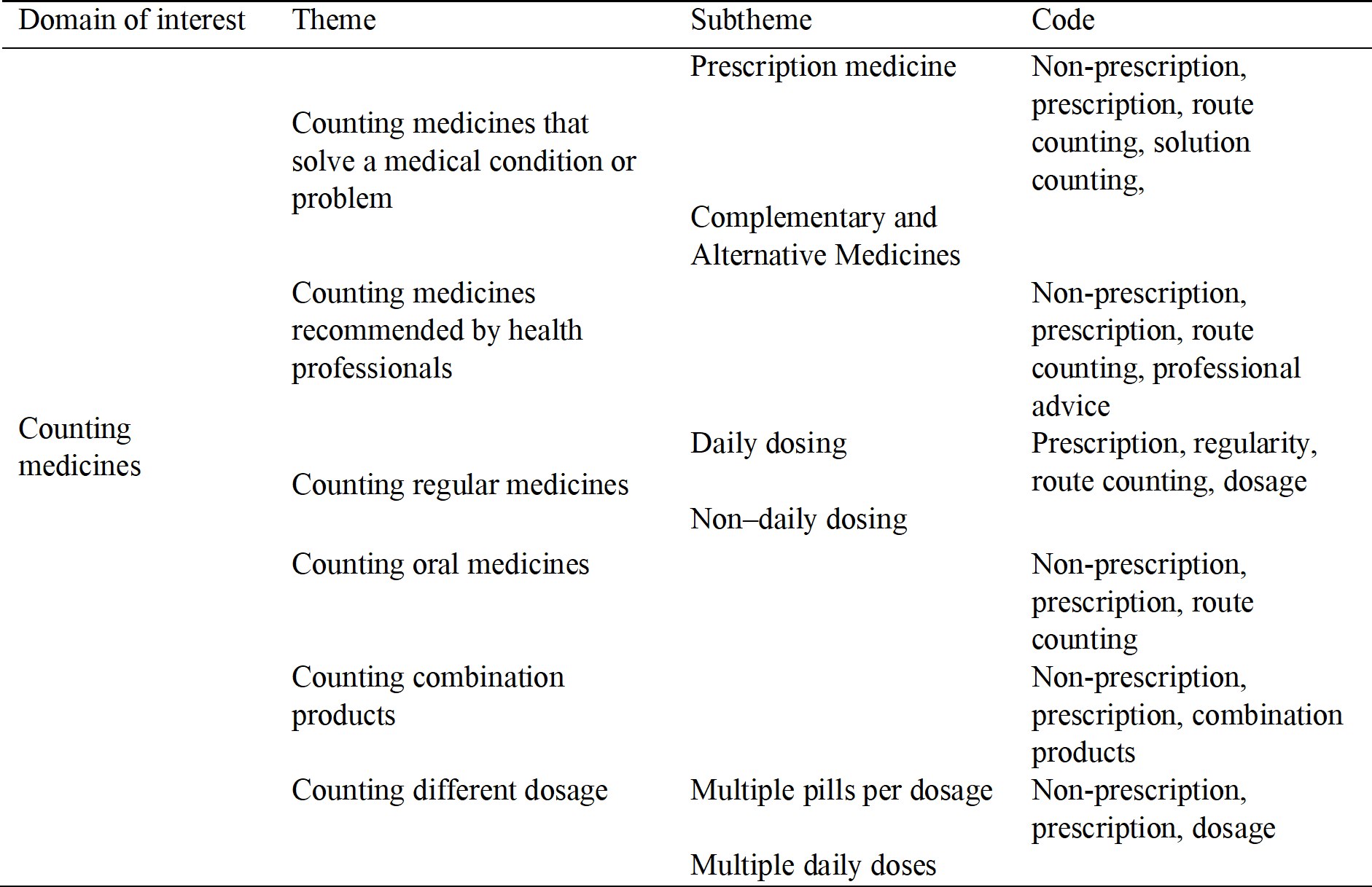

Supplement: Supplementary file 1 — Supplementary file1 (DOCX 1695 KB) [file 11096_2025_1890_MOESM1_ESM.docx]
